# Ulceroglandular Infection and Bacteremia Caused by *Francisella salimarina* in Immunocompromised Patient, France

**DOI:** 10.3201/eid2802.211380

**Published:** 2022-02

**Authors:** Aurélie Hennebique, Yvan Caspar, Max Maurin, Sandrine Boisset, Isabelle Pelloux, Maria Pilar Gallego-Hernanz, Christophe Burucoa, France Cazenave-Roblot, Chloé Plouzeau, Blandine Rammaert

**Affiliations:** Université Grenoble Alpes, Grenoble (A. Hennebique, Y. Caspar, M. Maurin, S. Boisset);; CHU Grenoble Alpes, Grenoble, France (A. Hennebique, Y. Caspar, M. Maurin, S. Boisset, I. Pelloux);; CHU de Poitiers, Poitiers, France (M.P. Gallego-Hernanz, C. Burucoa, F. Cazenave-Roblot, C. Plouzeau, B. Rammaert);; Université de Poitiers, Poitiers (C. Burucoa, F. Cazenave-Roblot, B. Rammaert)

**Keywords:** *Francisella salimarina*, *Francisella* species, opportunistic infections, France, bacteria, zoonoses

## Abstract

Although *Francisella tularensis* is a well-known, highly virulent bacterium that causes tularemia in humans, other *Francisella* species have been associated with sporadic human infections. We describe a human cutaneous infection with bacteremia caused by *F. salimarina*, a *Francisella* species recently identified from seawater and fishes, in an immunocompromised patient in France.

Although the taxonomy of the genus *Francisella* includes a wide diversity of species, only *F. tularensis* subspecies *tularensis* and *F. tularensis* subsp. *holarctica* cause the potentially life-threatening disease tularemia (*1*). Several *Francisella* spp., including *F. philomiragia*, *F. novicida*, *F. opportunistica*, and *F. hispaniensis*, are occasional opportunistic human pathogens; the other *Francisella* spp. are not associated with human infections (*1*). We describe a human infection caused by *F. salimarina*, recently identified from aquatic environments and fishes.

In June 2017, a 76-year-old man received a diagnosis of acute myelomonocytic leukemia and was admitted to Poitiers University Hospital (Poitiers, France). The patient lived in a small town 30 km from the Atlantic Ocean, had not travelled abroad recently, and had no pets. The day after admission, first-line chemotherapy of subcutaneous azacitidine was started for 7 days. After 3 days of chemotherapy, piperacillin/tazobactam was introduced for 5 days because of febrile aplasia. The patient was then discharged with an antibiotic prophylaxis (sulfamethoxazole/trimethoprim at 800 mg/160 mg 3×/wk). On July 26, the second azacitidine treatment was not administered because the patient again experienced febrile aplasia. Physical examination revealed skin lesions on 2 left-hand fingers that had appeared 3 weeks earlier. These lesions were erythematous and crusty but not purulent (Figure, panel A). They were associated with a left axillary lymphadenopathy. Antibiotic treatment with piperacillin/tazobactam and teicoplanin was started but was changed to imipenem/cilastatin and daptomycin after 5 days because of poor clinical response. Aerobic blood cultures performed at admission tested positive on July 31 and Gram stain showed a small gram-negative coccobacillus (Figure, panel B). Antibiotic treatment was changed to cefepime, administered for 3 days. No identification could be obtained by MALDI-TOF (matrix-assisted laser desorption/ionization time-of-flight) mass spectrometry (Vitek MS; bioMérieux, https://www.biomerieux.com). The strain was identified as a *Francisella* spp. by 16s rDNA amplification and sequencing. A cutaneous biopsy was performed because of persistent fever and worsening skin lesions in the patient; the same *Francisella* spp. strain was isolated. Doxycycline (100 mg 2×/d) was administered for 8 days, followed by sulfamethoxazole/trimethoprim, which led to apyrexia. 

**Figure:**
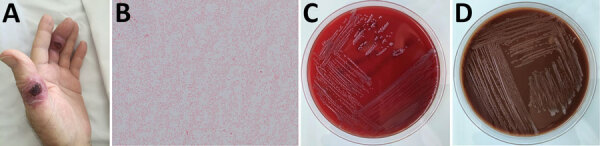
Skin ulcers and bacteremia caused by *Francisella salimarina* in an immunocompromised patient and isolated bacteria morphology, France. A) Skin lesion on 2 left-hand fingers. B) Small gram-negative coccobacillus isolated from blood and skin lesions (original magnification ×1,000). C) Growth on blood agar after 2 days of incubation at 35°C in 5% CO_2_. D) Growth on chocolate agar after 2 days of incubation at 35°C in 5% CO_2_.

The *Francisella* spp. strain (referred to as CHUGA-F75) was sent to the French National Reference Centre for *Francisella* for further characterization. The strain was strictly aerobic and grew well on chocolate agar supplemented with IsoVitaleX (bioMérieux), blood agar, and tryptic soy agar, yielding gray mucoid colonies after 24 h of incubation at 35°C in 5% CO_2_, but not on Drigalski agar (Figure, panels C, D). Biochemical testing revealed a positive oxidase, a weakly positive catalase, and a negative urease test. The strain was also halotolerant; it could grow in modified Mueller-Hinton broth with up to 8% NaCl. ISFtu2, Tul4, and type B real-time PCR tests, which detected most *Francisella* spp., *F. tularensis*, and *F. tularensis* subsp. *holarctica*, were all negative for DNA extracted from this strain (*2*,*3*). Species identification could not be obtained by using MALDI-TOF mass spectrometry, either with the routine database (MBT IVD Library DB-7171), the Biotox database (MBT SR Library; both from Bruker, https://www.bruker.com), or the French National Reference Centre for *Francisella* database containing *F. tularensis*, *F. novicida*, and *F. philomiragia* (*4*). Therefore, we performed whole-genome sequencing by using second and third next-generation sequencing platforms MiSeq (Illumina, https://www.illumina.com) and MinION (Oxford Nanopore Technologies, https://nanoporetech.com). Hybrid assembly of the sequencing data using Unicycler software on the Galaxy web platform (https://usegalaxy.org) enabled circularization of a 1,940,863 bp bacterial chromosome (Genbank accession no. CP076680). Whole-genome–based identification of the strain was assessed by using the Type Strain Genome Server (https://tygs.dsmz.de) (*5*). The CHUGA-F75 strain clustered in the same branch as the *F. salimarin*a SYSU SYW-1, the *F. marina* E95-16, and the *F. salina* TX07-7308 strains (Appendix Figure), probably representing the same species because of high genetic homology, although different species names have been published (*6*–*8*). Digital DNA-DNA hybridization >70%, average nucleotide identity >95%, and difference in percent guanine-cytosine content <1 percent between the CHUGA-F75 strain and the 3 *F*. *salimarina*, *F*. *marina*, and *F*. *salina* strains confirmed the CHUGA-F75 isolate belonged to the same species. Because the only validly published species name according to the International Code of Nomenclature of prokaryotes is *F. salimarina*, we identified CHUGA-F75 as *F. salimarina.* Using the broth microdilution method in cation-adjusted Mueller-Hinton broth as recommended by the Clinical and Laboratory Standards Institute, we found that the CHUGA-F75 strain was sensitive to gentamicin (MIC = 0.125 mg/L), doxycycline (MIC = 1 mg/L), and ciprofloxacin (MIC = 0.016 mg/L) and resistant to sulfamethoxazole/trimethoprim (MIC = 32 mg/L).

*F. marina* was described as responsible for systemic disease in fishes (*Lutjanus guttatus*, the cultured spotted rose snapper) in Central America, whereas 4 *F. salimarina* strains have been isolated from costal seawater in Guangdong Province, China, and 1 strain of *F. salina* has been grown from brackish seawater and seaweed off the coast of Galveston, Texas, USA (*6*–*8*). To our knowledge, these *Francisella* spp. were not responsible for human infection so far. This report, like previous descriptions of human infections caused by emergent *Francisella* spp., highlights that environmental or fish-related *Francisella* spp. could be responsible for opportunistic human infections resembling tularemia.

Appendix Additional information about ulceroglandular infection and bacteremia caused by *Francisella salimarina* in immunocompromised patient, France
