# Supplementary material for: Ulceroglandular Infection and Bacteremia Caused by Francisella salimarina in Immunocompromised Patient, France
Source: Emerg Infect Dis. 2022 Feb;28(2):465–7. doi: 10.3201/eid2802.211380 (PMC8798692; doi:10.3201/eid2802.211380)
Supplement: Appendix — Additional information about ulceroglandular infection and bacteremia caused by Francisella salimarina in immunocompromised patient, France [file 21-1380-Techapp-s1.pdf]

Article DOI: https://doi.org/10.3201/eid2802.211380

Ulceroglandular Infection and Bacteremia Caused by *Francisella salimarina* in Immunocompromised Patient, France

Appendix

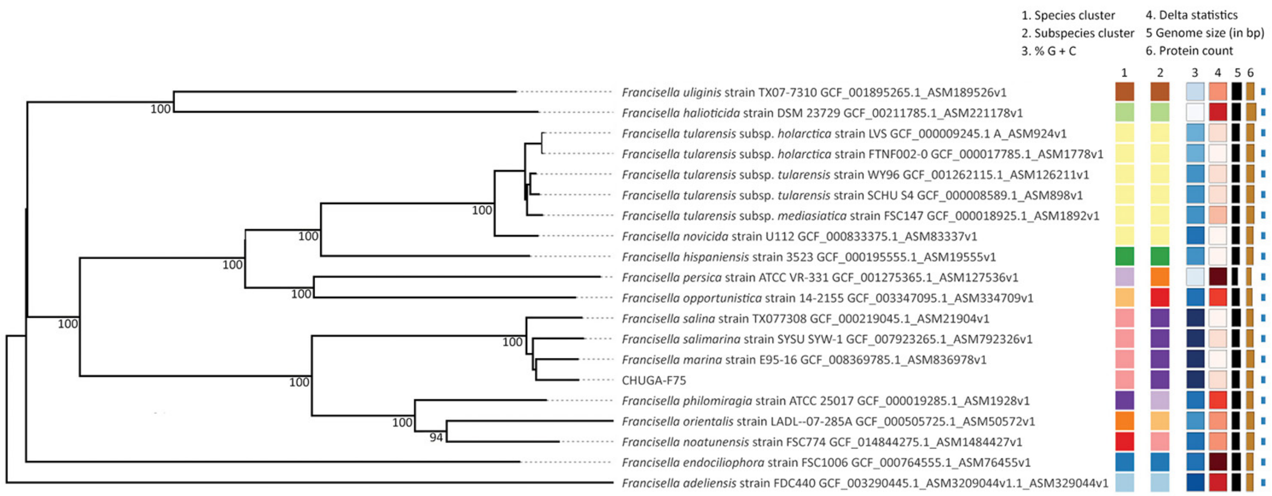

Appendix Figure. Genome-scale GBDP (Genome Blast Distance Phylogeny) tree of CHUGA-F75 strain of *Francisella* species, France. Whole genome–based identification of the strain was assessed by using the Type Strain Genome Server (https://tygs.dsmz.de).
